# Enhancing *S*-adenosyl-methionine catabolism extends *Drosophila* lifespan

**DOI:** 10.1038/ncomms9332

**Published:** 2015-09-18

**Authors:** Fumiaki Obata, Masayuki Miura

**Affiliations:** 1 Department of Genetics, Graduate School of Pharmaceutical Sciences, The University of Tokyo, 7-3-1 Hongo, Bunkyo-ku, Tokyo 113-0033, Japan; 2 CREST, Japan Agency for Medical Research and Development, 20F Yomiuri Shimbun Building 1-7-1 Otemachi, Chiyoda-ku, Tokyo 100-0004, Japan

## Abstract

Methionine restriction extends the lifespan of various model organisms. Limiting *S*-adenosyl-methionine (SAM) synthesis, the first metabolic reaction of dietary methionine, extends longevity in *Caenorhabditis elegans* but accelerates pathology in mammals. Here, we show that, as an alternative to inhibiting SAM synthesis, enhancement of SAM catabolism by glycine *N*-methyltransferase (Gnmt) extends the lifespan in *Drosophila*. Gnmt strongly buffers systemic SAM levels by producing sarcosine in either high-methionine or low-*sams* conditions. During ageing, systemic SAM levels in flies are increased. Gnmt is transcriptionally induced in a dFoxO-dependent manner; however, this is insufficient to suppress SAM elevation completely in old flies. Overexpression of *gnmt* suppresses this age-dependent SAM increase and extends longevity. Pro-longevity regimens, such as dietary restriction or reduced insulin signalling, attenuate the age-dependent SAM increase, and rely at least partially on Gnmt function to exert their lifespan-extending effect in *Drosophila*. Our study suggests that regulation of SAM levels by Gnmt is a key component of lifespan extension.

## Abstract (web summary)

Inhibiting the formation of *S*-adenosyl-methionine (SAM) increases worm but not fly lifespan. Here the authors show that humans and flies possess the SAM-consuming enzyme Gnmt, the activity of which is regulated by lifespan-extending interventions, and that knockdown of Gnmt extends fly lifespan.

## Graphical abstract

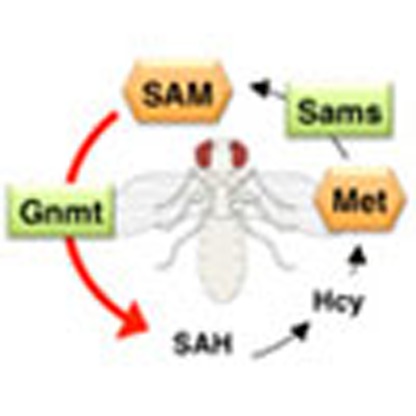

The most successful regimen to delay ageing and extend longevity is dietary restriction (DR), which was shown to increase lifespan from yeast to mammals^1^. However, a total reduction in calories may not be required, as reductions in specific macromolecules, such as amino acids or proteins, alone can also have an effect on organismal lifespan. In particular, methionine restriction (MR) has been shown to be sufficient for lifespan extension in yeast, worms, flies and rodents^2^,^3^,^4^,^5^. The evolutionally conserved enzyme methionine adenosyltransferase (MAT), also known as *S*-adenosyl-methionine synthase (Sams), first converts methionine into *S*-adenosyl-methionine, or SAM, a versatile methyl donor required for almost all methyltransferase (MTase) activity. Since various MTases catalyse the transfer of methyl groups to nucleic acids, proteins and metabolites, changes in SAM levels can result in altered epigenetic regulation, lipid metabolism or protein function^6^. Moreover, downstream metabolites of SAM include polyamines and cysteine, both of which are related to longevity by inducing autophagy^7^ and conferring oxidative stress resistance, respectively^8^. Therefore, SAM and/or its downstream metabolites, rather than methionine, seems to be the main effector of the MR-dependent increase in lifespan. Indeed, knockdown of *sams1* in *Caenorhabditis elegans*, which results in decreased SAM, extends longevity, possibly through a reduction in protein translation^9^,^10^. However, loss of MAT1A function in mice induced liver damage, which led to steatosis and eventually developed into hepatocellular carcinoma^6^. Human patients with chronic liver disease are reported to have decreased capacity to synthesize SAM^11^, suggesting that a reduction in SAM synthesis is harmful in humans.

The goal of our study was to determine whether alteration of SAM metabolism resulted in positive or negative effects on the *Drosophila* lifespan. We found that glycine *N*-methyltransferase (Gnmt) is a predominant regulator of SAM levels in *Drosophila*. Overexpressing Gnmt could rescue the age-dependent SAM increases and extend *Drosophila* lifespan. Our data demonstrate that enhanced SAM catabolism by Gnmt is a key factor for lifespan control.

## Results

### Knockdown of SAM synthase shortened *Drosophila* lifespan

**Figure 1:**
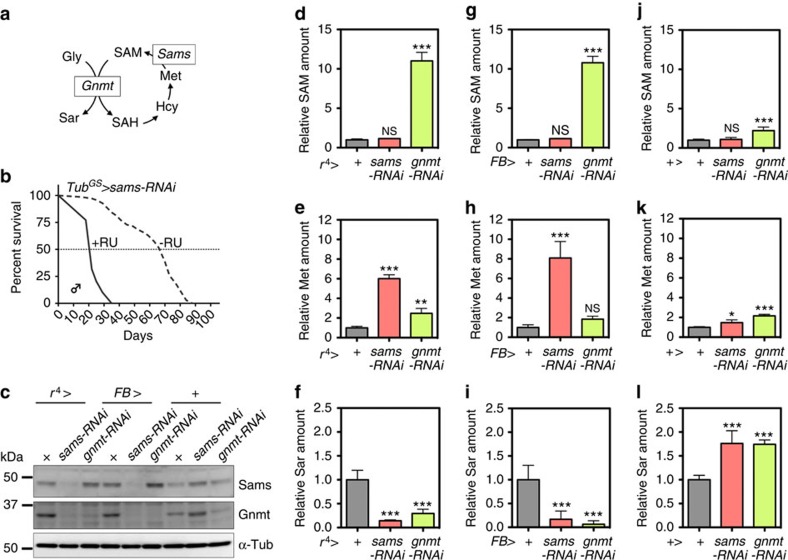
Gnmt is a predominant regulator of systemic SAM levels. (**a**) Schematic view of methionine metabolism in *Drosophila melanogaster*. Gly, glycine; Sar, sarcosine; Met, methionine; SAM, *S*-adenosyl-methionine; SAH, *S*-adenosyl-homocysteine; Hcy, homocysteine; Sams, *S*-adenosyl-methionine synthase; Gnmt, glycine *N*-methyltransferase. (**b**) Lifespan analysis of ubiquitous *sams*-RNAi male flies under 10% SY diet. In total, 200 μM RU486 (RU) is used to knock down *sams* after adult eclosion in *Tub*^*GS*^*>sams*-RNAi. Statistics, log-rank test, *P*<0.0001 (*N*=70 for −RU, *N*=79 for +RU). (**c**) Western blot analysis of Sams and Gnmt in day-5 male flies with either *sams*-RNAi or *gnmt*-RNAi driven by the fat-body drivers: *r*^*4*^*-Gal4* and *FB-Gal4*, or no driver. + Indicates UAS-only or Gal4-only controls. (**d**–**l**) UPLC–MS/MS analysis of SAM, methionine (Met) and sarcosine (Sar) levels in day-5 male flies with either *sams*-RNAi or *gnmt*-RNAi driven by the fat-body drivers (*FB-Gal4*, *r*^*4*^*-Gal4*) or no driver. + Indicates UAS-only or Gal4-only controls. Error bars represent mean±s.d. (*N*=4). Statistics, one-way analysis of variance with Bonferroni's multiple comparison test. *P<0.05, **P<0.01, ***P<0.001 from the biological replicates. NS, not significant.

*Drosophila melanogaster* has a single SAM synthase (*sams*) (Fig. 1a). We first knocked down *sams* to test whether it increases the lifespan in *Drosophila*, as reported in *C. elegans*. *UAS-sams*-RNA interference (RNAi) crossed with an ubiquitous driver, *da-Gal4*, showed developmental lethality, indicating that *sams* is an essential gene for proper development. Next, we checked the lifespan of male flies with adult-specific knockdown of *sams* by *Tub*^*GS*^*-Gal4*, which is the RU486-inducible binary expression system in the whole body. We were able to bypass the developmental lethality of *sams*-RNAi, and thus, we analysed the adult lifespan in the presence or absence of RU486. In contrast to *sams*-RNAi in *C. elegans*, we observed a significantly shortened lifespan in *Drosophila* (Fig. 1b).

It was possible that *sams*-RNAi had tissue-specific negative effects. Because methionine metabolism is active in the liver of mammals, we knocked down *sams* specifically in the fat body, a counterpart of the liver and white adipose tissue in *Drosophila*. We compared the lifespan of *FB>sams*-RNAi flies to UAS-only control flies, as *FB-Gal4*, but not *UAS-sams-RNAi*, was backcrossed in this experiment. Both the female and male lifespans were significantly decreased by knocking down *sams* in fat body (Supplementary Fig. 1a,b). Although this manipulation is not adult specific, as *FB-Gal4* is also expressed during the developmental stage, inhibiting SAM synthesis in *Drosophila* did not demonstrate positive effects on lifespan, raising a question about what causes the different outcomes between flies and worms.

### Gnmt is a dominant regulator of systemic SAM levels

We suspected that there were differences in the metabolic regulation of SAM. To find out how SAM metabolism was affected by *sams*-RNAi, we analysed the metabolites of methionine metabolism by ultra-performance liquid chromatography tandem mass spectrometry (UPLC–MS/MS) (Fig. 1c–l). Surprisingly, *sams*-RNAi in the fat body using two different fat-body *Gal4* drivers (*FB-Gal4* and *r*^*4*^*-Gal4*) did not result in a systemic SAM reduction (Fig. 1d,g), despite the marked decrease in Sams proteins (Fig. 1c). Methionine was drastically elevated, indicating that the synthesis of SAM from methionine was indeed inhibited (Fig. 1e,h). Fruit flies, mice and humans, but not worms, have Gnmt (http://www.genome.jp/kegg/) that catalyses the conversion of glycine to sarcosine (*N*-methyl-glycine) by methyl group transfer using SAM (Fig. 1a). Gnmt works as a regulator of SAM levels in metabolic organs such as liver in mammals or the fat body in *Drosophila*^6^,^12^. Knockdown of *gnmt* in fat body led to reduced Gnmt proteins (Fig. 1c), elevated SAM levels (Fig. 1d,g) and decreased Sar levels (Fig. 1f,i), although there is an unexpected slight increase in all three metabolites in negative-control flies (Fig. 1j–l). When methionine was added to food, the increase in SAM levels was limited, whereas the amount of sarcosine was increased in a dose-dependent manner (Supplementary Fig. 1c–e). Conversely, sarcosine levels significantly decreased in *sams* knockdown male flies (Fig. 1f,i), in contrast to negative-control flies (where they were increased for unknown reasons) (Fig. 1l), indicating that Gnmt maintains SAM levels by reducing SAM consumption. Interestingly, *sams*-RNAi in the fat body led to a reduction in Gnmt protein (Fig. 1c), which corresponded to a reduced amount of sarcosine, suggesting the existence of transcriptional or post-transcriptional regulation of Gnmt expression for maintaining SAM levels. Considering that *sams1*-RNAi resulted in a decrease in the amount of SAM in *C. elegans*^13^, the buffering of SAM levels by Gnmt may explain the different *sams*-RNAi phenotype. However, there are other possibilities, including differences in the knockdown efficiency of *sams* or the number of *sams* genes (four in *C. elegans*, but only one in *D. melanogaster*).

**Figure 2:**
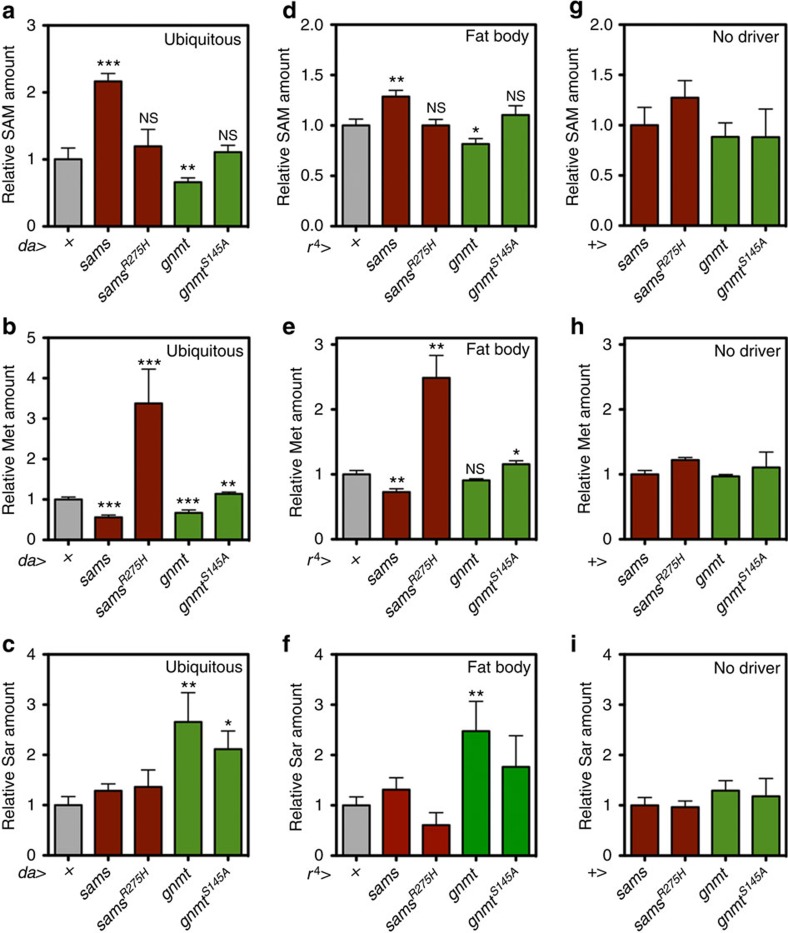
The effect of overexpression of Sams and Gnmt on metabolite levels. (**a**–**i**) UPLC–MS/MS analysis of SAM, Met and Sar levels in day-5 male flies with *sams*, *sams*^*R275H*^, *gnmt* and *gnmt*^*S145A*^ overexpression by ubiquitous (*da-Gal4*) or fat-body (*r*^*4*^*-Gal4*) drivers or no driver. + Indicates UAS-only or Gal4-only controls. Statistics, two-tailed unpaired *t*-test. Error bars represent mean±s.d. (*N*=5 for *da-Gal4*, *N*=3 for *r*^*4*^*-Gal4*, *N*=4 for no-driver control). *P<0.05, **P<0.01, ***P<0.001 from the biological replicates. NS, not significant.

We further analysed how these metabolites are affected by overexpressing either *sams* or *gnmt* ubiquitously or in a fat-body-specific manner (Fig. 2a–i; supplementary Fig. 2a). *sams* overexpression decreased Met and increased SAM. Introducing the R275H mutation, which was observed in several human hypermethioninemia patients who had a dominant *MAT1A*^*R264H*^ mutation^14^, led to increased Met without affecting SAM levels. This result resembled the *sams*-RNAi phenotype. MAT1A in mammals forms dimers (called MAT III) or tetramers (called MAT I). R264 is essential for dimerization, and the R264/R264H dimer is enzymatically inactive^14^. Thus, we considered that Sams^R275H^ may behave as a dominant-negative form in *Drosophila* as well. On the other hand, *gnmt* overexpression decreased SAM levels, although subtly, as well as methionine levels. The reason methionine was also reduced in *gnmt*-overexpressing male flies was unknown; however, we thought that the reduction in SAM accelerated SAM synthesis from methionine as a feedback mechanism for maintaining SAM levels. We also established a mutant version of a *gnmt* overexpression line by introducing a serine 145 to alanine substitution. Since S145A is located at the SAM-binding site of Gnmt^15^, we speculated that this mutation might result in reduced enzymatic Gnmt function. Indeed, the effect of *gnmt*^*S145A*^ overexpression on SAM and methionine was attenuated, although it was not complete (Fig. 2a–i; supplementary Fig. 2b–d).

### Gnmt overexpression increased longevity

**Figure 3:**
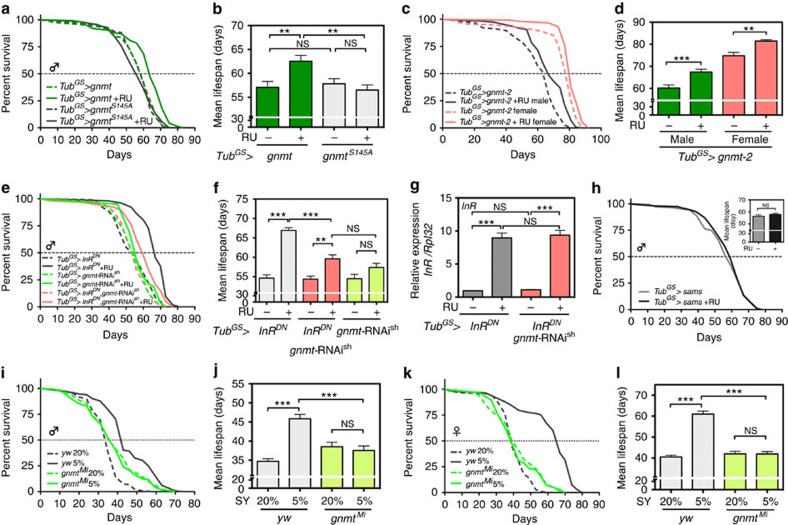
Gnmt is necessary for lifespan extension by reduced insulin signalling or dietary restriction. (**a**) Lifespan analysis of Gnmt-overexpressing male flies by *Tub*^*GS*^*-Gal4.* Statistics, log-rank test, *gnmt* versus *gnmt*+RU, *P*<0.0001 (*N*=117 for *gnmt*, *N*=118 for *gnmt*+RU). *gnmt*^*S145A*^ versus *gnmt*^*S145A*^+RU, *P*=0.7732 (*N*=111 for *gnmt*^*S145A*^, *N*=113 for *gnmt*^*S145A*^+RU). (**b**,**d**,**f**,**j**,**l**) Average lifespan. One-way analysis of variance (ANOVA) with Bonferroni's multiple comparison test was used for statistical analysis. (**c**) Lifespan analysis of *gnmt*-overexpressing male and female flies by *Tub*^*GS*^*-Gal4. UAS-gnmt* integrated on *attP2* site is used. Statistics, log-rank test, *gnmt-2* male versus *gnmt-2*+RU male, *P*<0.0001 (*N*=116 for each). *gnmt-2* female versus *gnmt-2*+RU female, *P*<0.0001 (*N*=116 for each). (**e**) Lifespan analysis of male flies overexpressing *InR*^*DN*^ (dominant-negative form of the insulin receptor), *gnmt*-RNAi^sh^ or both. Statistics, log-rank test, *InR*^*DN*^ versus *InR*^*DN*^+RU, *P*<0.0001 (*N*=158 for *InR*^*DN*^, *N*=154 for *InR*^*DN*^+RU). *gnmt*-RNAi^sh^ versus *gnmt*-RNAi^sh^+RU, *P*=0.0478 (*N*=80 for both). *InR*^*DN*^, *gnmt*-RNAi^sh^ versus *InR*^*DN*^, *gnmt*-RNAi^sh^+RU, *P*<0.0001 (*N*=142 for *InR*^*DN*^, *gnmt*-RNAi^sh^, *N*=154 for *InR*^*DN*^, *gnmt*-RNAi^sh^+RU). (**g**) qRT–PCR analysis of *InR* in day-7 adult male flies treated RU486 for 5 days. Error bars represent mean±s.e.m. (*N*=4). Statistics, one–way ANOVA with Bonferroni's multiple comparison test. (**h**) Lifespan analysis of male flies overexpressing *sams*. Statistics, log-rank test, *sams* versus *sams*+RU, *P*=0.6381 (*N*=135 for *sams*, *N*=143 for *sams*+RU). Inset, average lifespan. (**i**) Lifespan analysis of male *gnmt*^*Mi*^ flies under dietary restriction. Statistics, log-rank test, *yw* 20 versus 5%, *P*<0.0001 (*N*=134 for 20%, *N*=132 for 5%). *gnmt*^*Mi*^ 20 versus 5%, *P*=0.5904 (*N*=133 for 20%, 129 for 5%). (**k**) Lifespan analysis of female *gnmt*^*Mi*^ flies under dietary restriction. Statistics, log-rank test, *yw* 20 versus 5%, *P*<0.0001 (*N*=140 for 20%, *N*=133 for 5%). *gnmt*^*Mi*^ 20 versus 5%, *P*=0.9107 (*N*=134 for 20%, 136 for 5%). *P<0.05, **P<0.01, ***P<0.001 from the biological replicates. NS, not significant.

We then assessed whether SAM reduction in *gnmt*-overexpressing flies had a positive impact on lifespan. When *gnmt* was overexpressed using *Tub*^*GS*^*-Gal4*, we observed a slight but statistically significant increase in male lifespan (Fig. 3a,b). Several enzymes, including Gnmt, have functions other than enzymatic activity^16^, making us question whether Gnmt-induced longevity is dependent on its enzymatic activity. Overexpression of *gnmt*^*S145A*^ by *Tub*^*GS*^*-Gal4* did not show any significant effect on lifespan, confirming that enhancing enzymatic activity of Gnmt is necessary for lifespan extension (Fig. 3a,b). Unexpectedly, we did not observe the lifespan increase in female flies when we compared lifespans of *gnmt* with those of *gnmt*+RU (Supplementary Fig. 3a,b). However, there was a significant increase in *gnmt* when compared with *gnmt*^*S145A*^, even in the absence of RU. It is possible that leaky expression of the *Tub*^*GS*^ driver was enough to extend lifespan in females, or that the position (*attP40*) effect of *UAS-gnmt* could have different effects on males and females. Therefore, we also checked the effect of Gnmt overexpression on lifespan using a different *gnmt* overexpression line, which was inserted at the *attP2* site (*gnmt*-2). In line with this, we observed a significant increase in male and female lifespans (Fig. 3c,d), suggesting that Gnmt overexpression can be beneficial for both sexes.

### Gnmt is essential for lifespan-extending regimens

Genetic and pharmacological interventions targeting key metabolic pathways such as insulin/IGF-1 signalling (IIS) or the target of rapamycin (TOR) pathway also increase organismal lifespan^1^. Despite accumulating genetic studies using model organisms, our knowledge of the mechanisms underlying lifespan extension by DR or IIS/TOR inhibition is not complete. For example, inhibiting the IIS pathway exerts its effect on longevity through the transcription factor FoxO. However, the precise molecular targets and mechanism of FoxO-dependent lifespan increase have not been fully revealed. Interestingly, studies in *C. elegans* indicated *sams-1* expression is negatively regulated by *daf-16*, an orthologue of FoxO, implying that activation of FoxO under reduced IIS/TOR signalling or DR can extend longevity through repression of *sams-1* mesenger RNA^9^,^17^,^18^,^19^. In *Drosophila*, dFoxO transcriptionally induces *gnmt*^12^, but it did not repress *sams* at least at the whole-body level (Supplementary Fig. 3c), implying that Gnmt may contribute to IIS/FoxO-dependent lifespan extension. We observed in our experimental conditions that adult-specific overexpression of *InR*^*DN*^ in male flies significantly increased lifespan (Fig. 3e,f). As expected, we showed that knockdown of *gnmt* partially attenuated the lifespan extension of *Tub*^*GS*^*>InR*^*DN*^ (Fig. 3e,f), suggesting that Gnmt mediates lifespan extension under reduced IIS activity. This was not due to the dilution of Gal4 by introducing two UAS lines simultaneously, as we observed the same degree of *InR*^*DN*^ induction by RU treatment in both lines (Fig. 3g). Interestingly, *gnmt*-RNAi alone did not affect lifespan, indicating that increases in SAM do not have a negative impact on the lifespan (Fig. 3e,f). This was also supported by the data that *sams* overexpression in male flies did not affect the lifespan (Fig. 3h).

We also tested whether DR-induced longevity requires Gnmt. We used 20% SY diet as nutrient-rich food and 5% SY diet as DR diet. When compared with nutrient-rich condition, DR significantly extended the lifespans of both male and female flies in our lab conditions (Fig. 3i–l). Then, we subjected the *gnmt*^*Mi*^ loss-of-function mutant, which is the protein null mutant that we characterized previously and in which neither Gnmt protein nor sarcosine could be detected^12^, to DR lifespan analysis. Although *gnmt*^*Mi*^ has a different genetic background since it showed lethality when backcrossed to *w*^*1118*^, we found that DR-induced longevity was not observed in *gnmt*^*Mi*^ mutant (Fig. 3i–l), suggesting the possibility that Gnmt-dependent SAM catabolism mediates DR longevity. In addition, *gnmt* expression is positively regulated by the oxidative and xenobiotic stress-responsive factor CncC/Nrf2 (Supplementary Fig. 3d–h)^20^, which is also suggested as a mediator of DR longevity^21^, indicating that Gnmt is a common downstream target for longevity pathways.

### SAM levels increase during ageing despite Gnmt induction

**Figure 4:**
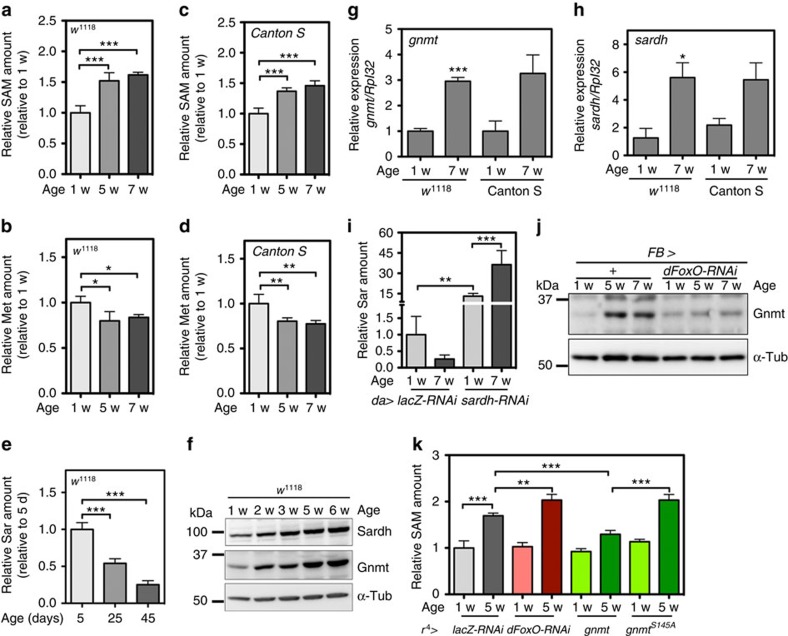
SAM levels increased during ageing in spite of induced *gnmt* expression by dFoxO in fat body. (**a**–**d**) UPLC–MS/MS analysis of SAM and Met levels in wild-type male flies during ageing. Error bars represent mean±s.d. (*N*=4). Statistics, one-way analysis of variance (ANOVA) with Bonferroni's multiple comparison test. (**e**) UPLC–MS/MS analysis of sarcosine levels in male *w*^*1118*^ during ageing. Error bars represent mean±s.d. (*N*=3). Statistics, one-way ANOVA with Bonferroni's multiple comparison test. (**f**) Western blot analysis of Sardh and Gnmt during ageing in male *w*^*1118*^. (**g**,**h**) qRT–PCR analysis of *gnmt* and *sardh* in young (1 week old) and old (7 weeks old) wild-type male flies. Error bars represent mean±s.e.m. (*N*=5 for 1 w *w*^*1118*^, *N*=6 for 7 w *w*^*1118*^, *N*=3 for Canton S). Statistics, two-tailed unpaired *t*-test 1 versus 7 w. (**i**) UPLC–MS/MS analysis of sarcosine levels in young and old male flies with *lacZ*-RNAi or *sardh*-RNAi. Error bars represent mean±s.d. (*N*=4). Statistics, One-way ANOVA with Bonferroni's multiple comparison test. (**j**) Western blot analysis of Gnmt in *dFoxO* knockdown flies.+ Indicates Gal4-only controls. (**k**) UPLC–MS/MS analysis of SAM levels in young and old male flies with *lacZ*-RNAi, *dFoxO*-RNAi, *gnmt* or *gnmt*^*S145A*^. Error bars represent mean±s.d. (*N*=4). Statistics, one-way ANOVA with Bonferroni's multiple comparison test. w, week. *P<0.05, **P<0.01, ***P<0.001 from the biological replicates.

Although Gnmt overexpression extended longevity, and Gnmt is required for DR-induced longevity, it is still unknown whether Gnmt activity and SAM metabolism changes during physiological ageing. We quantified Met and SAM in two different wild-type strains analysing both young and aged male flies. Met levels decreased during ageing, whereas SAM levels increased (Fig. 4a–d). We also found that the amount of sarcosine decreased during ageing (Fig. 4e), suggesting Gnmt activity to buffer SAM declines in an age-dependent manner. However, the expression of Gnmt was induced in aged male flies, as determined by quantitative real-time PCR (qRT–PCR) and western blotting using whole-body homogenates (Fig. 4f,g; Supplementary Fig. 4a). Sarcosine levels are negatively regulated by sarcosine dehydrogenase (sardh)^12^, and Sardh was also induced transcriptionally during ageing (Fig. 4f,h). Interestingly, when *sardh* expression was knocked down, sarcosine levels were high in young male flies and further induced by ageing (Fig. 4i), suggesting that total Gnmt activity is indeed increased during ageing. Gnmt was increased in the fat body, because fat-body-specific knockdown of *gnmt* abrogated elevation of Gnmt in whole-body homogenates (Supplementary Fig. 4b). This increase was dependent on dFoxO (Fig. 4j; Supplementary Fig. 4c). Induction of Gnmt expression during ageing might be an adaptive response against an increase in SAM levels. Indeed, when *dFoxO* was knocked down in the fat body, SAM levels in aged male flies were further elevated (Fig. 4k). Therefore, we concluded that the dFoxO–Gnmt pathway is activated, but not sufficiently for complete suppression of the SAM increase in aged flies. Importantly, overexpression of *gnmt*, but not *gnmt*^*S145A*^, inhibited the age-dependent SAM increase (Fig. 4k; Supplementary Fig. 4d,e), indicating that Gnmt-induced lifespan extension is caused by suppression of the age-dependent increase in SAM.

### SAM levels are maintained under pro-longevity regimens

**Figure 5:**
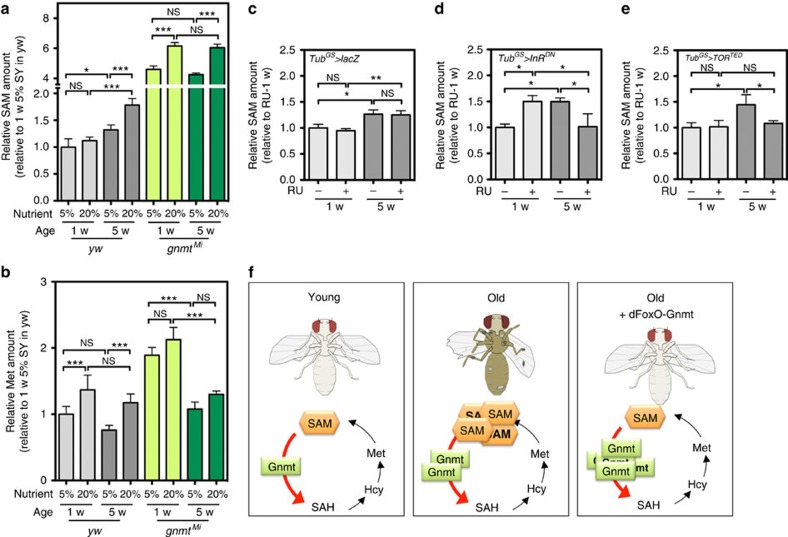
Age-dependent SAM increase is attenuated by lifespan-extending regimens. (**a**,**b**) UPLC–MS/MS analysis of SAM and Met levels in *yw* and *gnmt*^*Mi*^ young (1 weeks old) or old (5 weeks old) male flies maintained under dietary restriction or a nutrient-rich condition. Error bars represent mean±s.d. (*N*=3–4). Statistics: one-way analysis of variance (ANOVA) with Bonferroni's multiple comparison test. (**c**–**e**) UPLC–MS/MS analysis of SAM levels in *lacZ*-, *InR*^*DN*^or *TOR*^*TED*^-overexpressing male flies at young (1 week old) or old (5 weeks old) stages. Error bars represent mean±s.d. (*N*=3–4). Statistics: one-way ANOVA with Bonferroni's multiple comparison test. (**f**) Schematic view of the relationship between SAM metabolism and longevity control. Compared with young flies, SAM levels are increased despite Gnmt induction in old flies. Strengthening the dFoxO–Gnmt axis rescues age-dependent SAM increases and extends lifespan. *P<0.05, **P<0.01, ***P<0.001 from the biological replicates. NS, not significant.

Since Gnmt is required for DR-dependent lifespan extension, we analysed how DR altered systemic SAM levels. We analysed young (1 weeks (w)) and aged (5 w) male flies from two wild-type strains as well as a *gnmt* mutant strain maintained on a DR- (5% SY) or nutrient-rich (20% SY) diet. Since the 20% SY diet contains much Met, young flies maintained on 20% SY diet showed a slight increase in Met levels; however, changes in SAM levels were not statistically significant (Fig. 5a,b; Supplementary Fig. 5a,b), suggesting that Gnmt buffered SAM increase. Indeed, the lack of regulation by *Gnmt* resulted in increased SAM in male flies on the 20% SY diet compared with that of files on the 5% SY diet (Fig. 5a). As previously mentioned, Met levels tended to decrease with ageing in all three genotypes, but this phenotype was not affected by the diet (Fig. 5b; Supplementary Fig. 5b). In contrast, an age-dependent increase in SAM levels was suppressed by DR (Fig. 5a; Supplementary Fig. 5a). The *gnmt* mutants showed high SAM levels in young flies, but this was not further elevated by ageing (Fig. 5a), suggesting that a threshold for SAM increases exists in aged flies.

In addition to DR, we checked whether reduced IIS activity affected systemic SAM levels by analysing *Tub*^*GS*^*>InR*^*DN*^. Compared with control flies (*Tub*^*GS*^*>lacZ*), in which SAM was increased during ageing regardless of RU486 treatment (Fig. 5c), SAM levels were rather significantly decreased in *Tub*^*GS*^*>InR*^*DN*^ old male flies upon RU486 treatment than that in the control flies (old flies without RU486 or young flies with RU486), although the reason behind the RU486-induced SAM elevation in *Tub*^*GS*^*>InR*^*DN*^ young flies is unknown (Fig. 5d). Interestingly, an age-dependent SAM increase was also rescued by overexpressing the dominant-negative form of the TOR (*Tub*^*GS*^*>TOR*^*TED*^) (Fig. 5e). These data suggested that the suppression of SAM increases in aged flies was a common mechanism underlying lifespan extension by DR or reduced IIS/TOR pathway.

## Discussion

Our study indicates that the enhancement of SAM catabolism by Gnmt is an essential component for lifespan extension (Fig. 5f). Although Gnmt is transcriptionally induced during ageing at a site downstream of dFoxO activity in the fat body, this seemed to be insufficient to maintain SAM levels in aged flies. The reason behind the increase in SAM during ageing has yet to be elucidated; however, strengthening Gnmt activity attenuates the elevation of SAM and, importantly, extends longevity. Moreover, our data implied that DR and reduced IIS signalling (probably TOR and CncC as well) commonly target SAM metabolism to extend lifespan by inducing Gnmt. In humans, whether SAM levels increase in an age-dependent manner remains unknown, since only a few studies have tested this. However, one report suggested that serum SAM levels were higher in older individuals than in middle-aged individuals, at least in some populations^22^.

In our experimental conditions, *sams*-RNAi resulted in shorter lifespans. If present in excess, Met is a toxic compound in *Drosophila*^4^. It is therefore possible that hypermethioninemia in *sams* knockdown flies, the MAT1A knockout mice and patients with MAT1A deficiency causes adverse health effects^4^,^23^. However, whether *sams1*-RNAi in *C. elegans* results in the accumulation of methionine is unknown. Unexpectedly, loss of Gnmt function and subsequent SAM elevation did not have a negative effect on lifespan. The fact that the correlation of SAM levels and lifespan is not bidirectional implies a threshold in SAM levels that modulate organismal lifespan. One explanation is the biochemical character (for example, Km) of methyltransferases or other enzymes related to SAM-dependent metabolic pathways such as polyamine biosynthesis, methionine salvage pathway or trans-sulfuration pathway (TSP), as excess SAM does not always lead to elevated methylation or increased downstream metabolites.

The fact that Gnmt overexpression increases *Drosophila* lifespan suggests that decreases in SAM (and Met) and/or increase in SAM catabolites have a positive effect on longevity. For example, the acceleration of SAM catabolism by Gnmt may enhance the TSP, which will increase anti-oxidative capacity by upregulating cysteine, taurine and glutathione synthesis. In addition, TSP is critical for producing hydrogen sulfide, H_2_S, which is suggested to be the mediator of DR-induced benefits in both hepatic damage from ischaemia/reperfusion in mice and longevity in worms^24^. A study in *Drosophila* also suggests that TSP mediates DR-induced longevity^25^. Therefore, TSP or H_2_S might represent an underlying mechanism for Gnmt-dependent lifespan extension. In the *sams*-overexpressing flies in our study, SAM and probably downstream metabolites are increased. However, we did not observe any effect on lifespan in these flies, suggesting that not only enhancing SAM catabolism but also reducing SAM under the threshold is required for lifespan extension. In contrast, Gnmt overexpression reduces SAM and simultaneously enhances the generation of SAH and downstream metabolites. Whether reduction of SAM without enhancing SAM catabolism is sufficient for lifespan extension is not known, although it is suggested by the fact that *sams*-RNAi in worms can extend lifespan. Since lifespan represents a total sum of both positive and negative effect of different pathways, it is difficult to pinpoint the SAM-related pathway(s) essential for lifespan control, until we elucidate how each component affects lifespan.

It is also possible that SAM amount in host cells is recognized as a hallmark of nutrition availability. Thus, SAM reduction triggers the ‘fasting' response. For example, in yeast, nutrient poor diet induced autophagy, which was inhibited by methionine at least partially through the regulation of SAM-dependent PP2A methylation by *ppm1* methyltransferase^26^. Autophagy, induced by MR, was reported to be a direct cause of lifespan extension^3^, suggesting that SAM reduction-induced autophagy extends longevity, although no orthologue of *ppm1* is found in *Drosophila*. SAM-dependent transmethylation, including ribosomal RNA methylation, that affects lifespan through modulating translation^27^ is another possible connection between SAM and longevity. The exact molecular mechanisms behind the SAM effect on lifespan need to be investigated.

Ames dwarf mice are long-lived mutants that have defects in the production of growth hormone (GH) with consequent reductions in IGF-1 levels. Interestingly, Ames dwarf mice also show elevated GNMT expression and activity in addition to reduced SAM levels in their liver^28^,^29^. Administration of GH to Ames dwarf reduced GNMT activity while GH receptor knockout mice showed increased GNMT expression, indicating that GH signalling negatively regulates GNMT^30^. Although the contribution of GNMT in longevity was not studied, MR did not further extend lifespan in Ames dwarf mice, suggesting that altered methionine metabolism is responsible for longevity in these animals^31^. *gnmt* is one of seven genes commonly upregulated under DR (or resveratrol treatment) conditions in flies and mice^32^, further demonstrating that the positive effects of enhanced *gnmt* activity on longevity in mammals is conserved.

## Methods

### Fly stocks

Flies were reared on a standard diet containing 4% cornmeal, 4% baker's yeast (Oriental Yeast), 10% glucose and 0.8% agar with propionic acid and nipagin at constant 25 °C, 60% humidity under 12–12-h light–dark conditions. For most biochemical analysis other than lifespan study, all flies were collected within 2 days after adult eclosion and maintained for 5 days with free access to food and mating for adult maturation, unless otherwise stated.

*w*^*1118*^, *yw* and Canton S were utilized as wild-type strains. *UAS-gnmt*-RNAi^sh^ were generated in our previous study^12^. *Tub*^*GS*^*-Gal4* was kindly provided by S. Pletcher. *UAS-CncC* was kindly provided by D. Bohmann. *r*^*4*^*-Gal4*, *UAS-InR*^*DN*^ (K1409A), *UAS-dFoxO*-RNAi (HMS00422) and *UAS-dFoxO* were obtained from the Bloomington *Drosophila* stock center. *UAS-gnmt*-RNAi (v25983), *UAS-sams*-RNAi (v103143) and *UAS-keap1*-RNAi (v107052) were obtained from the Vienna *Drosophila* resource center. *da-Gal4*, *FB-Gal4*, *Mef2-Gal4*, *pxn-Gal4*, *NP1-Gal4*, *elav-Gal4*, *UAS-gnmt*-RNAi and *UAS-lacZ-*RNAi were all backcrossed at least six generations into *w*^*1118*^. *yw;gnmt*^*Mi*^ was maintained on its original genetic background, because it shows lethality when backcrossed onto *w*^*1118*^. Thus, we used the *yw* line as a control. *UAS-gnmt*, *UAS-gnmt*^*S145A*^, *UAS-sams* and *UAS-sams*^*R275H*^ were generated as described below. For overexpression of metabolic enzymes in fat body, *r*^*4*^*-Gal4* was used because it is stronger than *FB-Gal4.* For most biochemical analyses of adult male flies, we crossed the same batch of *Gal4* virgin females (once mixed after virgin collection, and then separated into each group) with different males including wild-type or *lacZ* lines as control. UAS-only controls were also analysed for some experiments, which we believe is essential.

### Construction and generation of transgenic flies

We established *gnmt* overexpression lines by phiC31-integrase-mediated transgenesis as follows. For making *UAS-gnmt*, a PCR-amplified 870-bp fragment of the full-length complementary DNA (cDNA)^12^ was reinserted into the *pUAS-attB* vector by *Eco*RI/*Xho*I, and it was then inserted into the attP40 site using phiC31-mediated transgenesis (Best Gene). For *UAS-gnmt*^*S145A*^, we subcloned full-length *gnmt* cDNA once into the *pBSSK* vector by *Eco*RI/*Xho*I and introduced the TCC→GCA mutation by PCR-based site-directed mutagenesis using the following primers: 5′-GGCAACGCATTTGCCCACTTGATGGAC-3′, and 5′-GGCAAATGCGTTGCCCAAGCAAATGAC-3′, followed by reinsertion into the *pUAS-attB* vector. For *UAS-sams*, a 1.2-kbp cDNA *sams* fragment was amplified by PCR from total RNA of adult *w*^*1118*^ using the following primers: 5′-GAATTCATGCCGCAAAAGAC-3′, and 5′-CTCGAGTCAGTTGTCAATCTCC-3′ and cloned into the *pT7* vector. *sams* cDNA was amplified by PCR using *pT7-sams* as template and the primers: 5′-GAAGATCTATGCCGCAAAAGAC-3′, and 5′-GGGCTCGAGTCAGTTGTCAATCTCC-3′ and subcloned once into the pUAST-3xFlag vector. The vector was then sequenced. Full-length, 1.2-kbp *sams* cDNA without a Flag tag was digested by *Bgl* II/*Xho*I and subcloned into the *pUAST-attB* vector. For UAS-*sams*^*R275H*^, *sams* cDNA was subcloned once into the *pBSSK* vector and a AGA→CAC mutation was introduced by PCR-based site-directed mutagenesis using the following primers: 5′-CTTACAGGACACAAAATCATTGTAGATACT-3′, and 5′-AATGATTTTGTGTCCTGTAAGCCCAGCGTC-3′. Transgenes were inserted into the *attP40* site on the second chromosome or *attP2* site on the third chromosome using phiC31-mediated transgenesis (Best Gene) and maintained as homozygous lines without crossing with balancer lines.

### Fly diet

For DR experiments, we prepared a 20% SY diet and 5% SY diet^25^. The 20% SY diet contains 20% baker's yeast (Oriental Yeast), 20% sucrose (Wako), and 1.5% agar (Kishida Chemical) with propionic acid and nipagin. The 5% SY diet contains 5% baker's yeast (Oriental Yeast), 5% sucrose (Wako) and 1.5% agar (Kishida Chemical) with propionic acid and nipagin. For making these diet, first yeast and sucrose were dissolved in water and boiled for 15 min to kill the living yeast completely. This mixture was added into agar, which was dissolved in water with 10-min heating in the different pot. After that, propionic acid and nipagin were added directly into the diet. For the gene switch (GS) system, 10% SY diet was prepared and RU486 (Sigma) was added into the 10% SY diet at a final concentration of 200 μM to drive gene expression. For the high-Met diet, we added 1, 2 or 5 ml of 100 mM stock methionine solution in a 100-ml standard diet.

### Lifespan analysis

To control environmental effects during development, we crossed the same number of female and male flies in the same period on a standard diet. Newly hatched flies were collected within, at most, 2 days, and then they were kept for 2 days under the mixed sex condition. Then, males and females were separated and grouped into 20 flies per vial. Flies were transferred to fresh food every 3 or 4 days, and at that time, we counted the number of dead flies.

### Western blotting and quantitative RT–PCR analysis

For western blot analysis, five adult male flies were homogenized in PBS supplemented with 0.1% SDS, 10 mM dithiothreitol and 1 × protease inhibitor cocktail (Roche). Samples were boiled for 5 min at 98 °C after mixing with 6 × Laemmli sample buffer. Whole-body proteins corresponding approximately to 0.15 fly were subjected to SDS–polyacrylamide gel electrophoresis and transferred onto polyvinylidene difluoride membrane. After 30 min blocking with 5% skimmed milk, membranes were incubated with the 1st antibody at 4 °C overnight. Anti-Gnmt antibody (1:3,000) and anti-Sardh antibody (1:1,500) were generated previously^12^. Anti-Sams antibody (1:1,000) was produced in guinea pigs using a synthetic peptide corresponding to the C terminus 382–400 of *Drosophila* Sams as an antigen. For loading control, anti-α-tubulin monoclonal antibody (1:3,000, Sigma DM1A) was used. Representative images were shown from at least two independent experiments with reproducible results. Full blot images for main figures are shown in Supplementary Fig. 6. For qRT–PCR, total RNA was purified from five adult males using the Qiazol and RNeasy micro kit (Qiagen). cDNA was made from 100 or 200 ng DNase-treated total RNA by the Takara PrimeScript RT Reagent Kit with gDNA Eraser. Quantitative PCR was performed using Takara Premix Ex Taq II (Tli RNaseH Plus) and the Light Cycler 480 system (Roche). For internal controls, *Rpl32* was predominantly used, but *RNA pol II* was also checked to rule out the possibility that changes in target gene expression resulted from altered expression of internal controls. Primer sequences are available in Supplementary Table 1.

### Measurement of metabolites by LC–MS/MS

Methionine, SAM and sarcosine levels were measured by UPLC equipped with tandem MS, TQD (UPLC–MS/MS, Waters)^12^. Briefly, five adult males were homogenized in 50% methanol, deproteinized by acetonitrile and evaporated completely. Pellets were solubilized in 10 mM HCl followed by filtration using 0.22-μm polyvinylidene difluoride filters (Millipore). Samples were mixed in equal volume of 50 mM Tris-HCl pH 8.8 with 100 μM dithiothreitol for Met and SAM measurements. For the sarcosine measurement, samples were derivatized after filtration by the AccQ-Tag Ultra Derivatization Kit (Waters). Samples were subjected to UPLC system with Acquity UPLC BEH C18 column. Separated solutions were ionized by electrospray ionization in positive-ion mode (ESI+) and detected by TQD with the following *m*/*z* transitions: Methionine, 149.98>132.90, SAM, 399.20>250.00 and derivatized sarcosine, 260.00>171.50.

Because absolute metabolite amounts can easily vary between experiments due to technical (for example, machine conditions) as well as biological (for example, season, food, genotype and so on) conditions, we always prepared a control sample in the same analytical round to compare the quantities as ratios relative to the control. To analyse the metabolites precisely, the quality of parents, the developmental environment and sampling time (to avoid circadian changes) were carefully controlled, and each graph was constructed from the same round of analysis.

## Additional information

**How to cite this article:** Obata, F. & Miura, M. Enhancing *S*-adenosyl-methionine catabolism extends *Drosophila* lifespan. *Nat. Commun.* 6:8332 doi: 10.1038/ncomms9332 (2015).

## Supplementary Material

Supplementary Information Supplementary Figures 1-6 and Supplementary Table 1.
