# Supplementary material for: Enhancing S-adenosyl-methionine catabolism extends Drosophila lifespan
Source: Nat Commun. 2015 Sep 18;6:8332. doi: 10.1038/ncomms9332 (PMC4595730; doi:10.1038/ncomms9332)
Supplement: Supplementary Information — Supplementary Figures 1-6 and Supplementary Table 1. [file ncomms9332-s1.pdf]

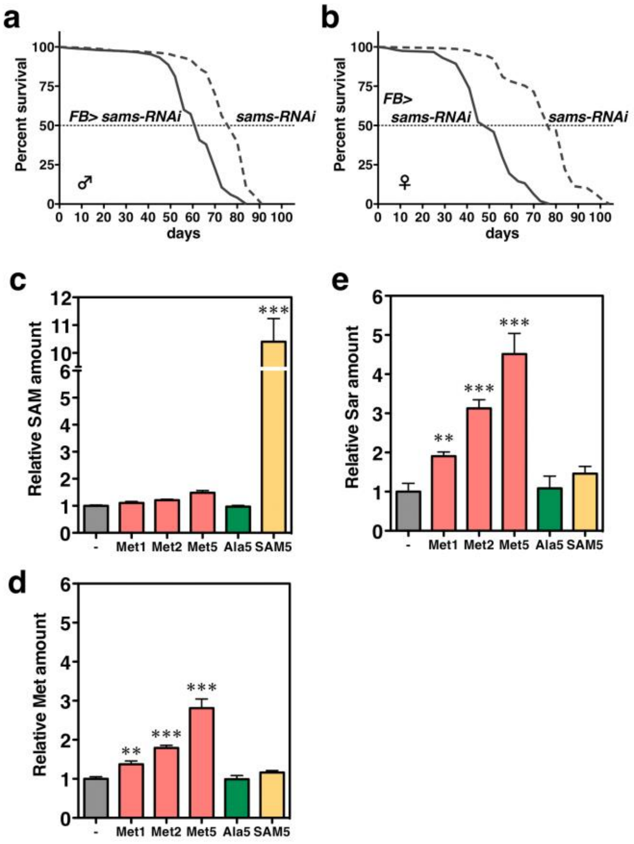

Supplementary Figure 1. The lifespan curve of *sams*-RNAi in fat body and buffering system of SAM after methionine feeding

(a,b) Male and Female lifespan of fat body-specific *sams*-RNAi flies (solid line, *FB>sams*-RNAi). *sams*-RNAi crossed to w^{1118} is used as a negative control (dotted line, *sams*-RNAi). Statistics, Male: Log-rank test, *P*< 0.0001 (N=160 for *sams*-RNAi, N=150 for *FB>sams*-RNAi). Female: Log-rank test, *P*< 0.0001 (N=158 for *sams*-RNAi, N=159 for *FB>sams*-RNAi). **(c-e)** Methionine (Met), SAM, and Sarcosine (Sar) levels in day-10 male flies maintained for 5 days on a diet containing an additional 1, 2, or 5 mM methionine, 5 mM Alanine, or 5mM SAM. High SAM levels were observed after SAM feeding, but it never lead to elevation of Sar (in contrast to Sar elevation in Met feeding), suggesting that SAM is not penetrated into tissues, at least fat body cells. Error bars represent mean ± SD (N=4). Statistics, One way ANOVA with Bonferroni's Multiple Comparison Test. For this and the following bar graphs, *p<0.05, **p<0.01, ***p<0.001 from the biological replicates.

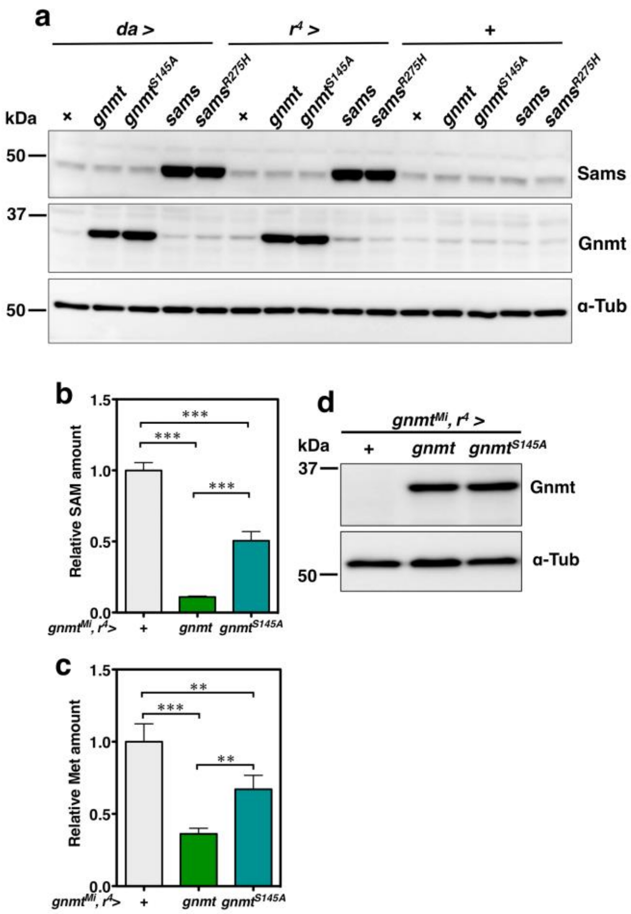

Supplementary Figure 2. The effect of overexpression of *gnmt* and *sams*

(a) Western blot analysis of Sams and Gnmt for overexpression lines. **(b-d)** Rescue of *gnmt* null mutant by overexpressing *gnmt* in fat body. **(b,c)** SAM and Met levels in flies overexpressing *gnmt* or *gnmt*^{S145A} in the fat body of *gnmt*^{Mi}. Error bars represent mean ± SD (N=4). Statistics, One way ANOVA with Bonferroni's Multiple Comparison Test. **(d)** Western blot analysis of Gnmt. All data were analyzed from day five-male flies.

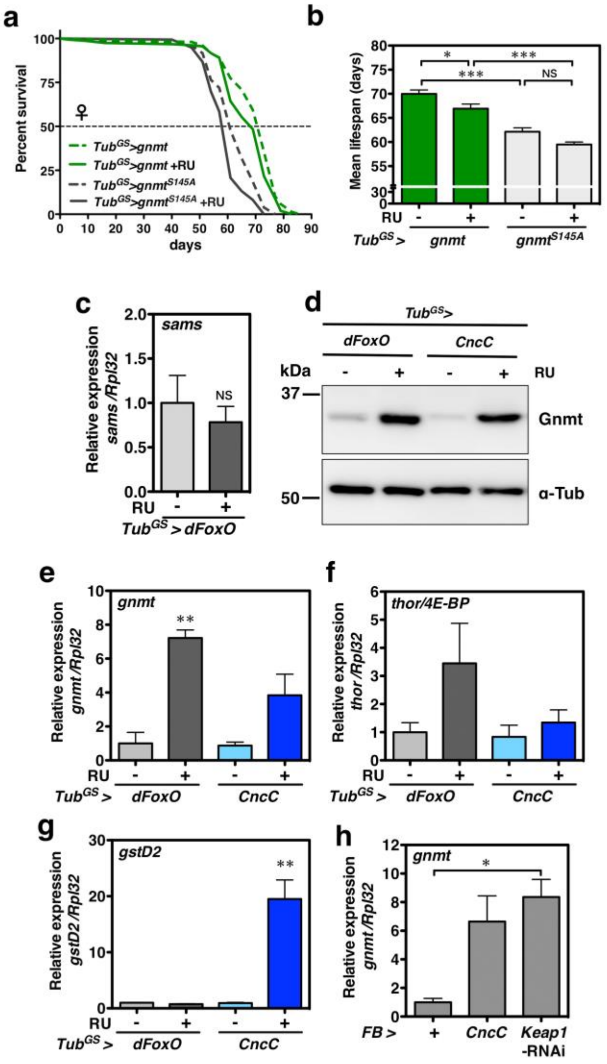

Supplementary Figure 3. Gnmt is regulated transcriptionally by dFoxO and CncC

(**a**) Lifespan analysis of Gnmt overexpressing female flies by *Tub^{GS}-Gal4*. Statistics, Log-rank test, *gnmt* vs *gnmt* +RU, *P*< 0.0001 (N=157 for *gnmt*, N=159 for *gnmt*+RU). *$gnmt^{S145A}$* vs *$gnmt^{S145A}$* +RU, *P*=0.0086 (N=155 for *$gnmt^{S145A}$*, N=157 for *$gnmt^{S145A}$* +RU). (**b**) Average lifespan. One way ANOVA with Bonferroni's Multiple Comparison Test was used for statistical analysis. **(c)** qRT-PCR analysis of *sams* expression in *Tub^{GS}>dFoxO* day-seven adult male flies treated RU486 for five days. Error bars represent mean ± SEM (N=3). Statistics, two-tailed Unpaired *t*-test. **(d)** Western blot analysis of Gnmt in *Tub^{GS}>dFoxO* or *Tu^{GS}>CncC* day-seven male flies treated with RU486 for five days. **(e-g)** qRT-PCR analysis of *gnmt*, *thor/4E-BP* (FoxO target gene), and *gstD2*

(CncC target gene) in *Tub*^{GS}*>dFoxO* or *Tub*^{GS}*>CncC* day-seven male flies treated with RU486 for five days. Error bars represent mean ± SEM (N=3 for dFoxO, N=4 for CncC). Statistics, two-tailed Unpaired *t*-test -RU vs +RU. **(h)** qRT-PCR analysis of *gnmt* in male flies overexpressing *CncC* or *Keap1*-RNAi in the fat body. Error bars represent mean ± SEM (N=3). Statistics, One way ANOVA with Bonferroni's Multiple Comparison Test.

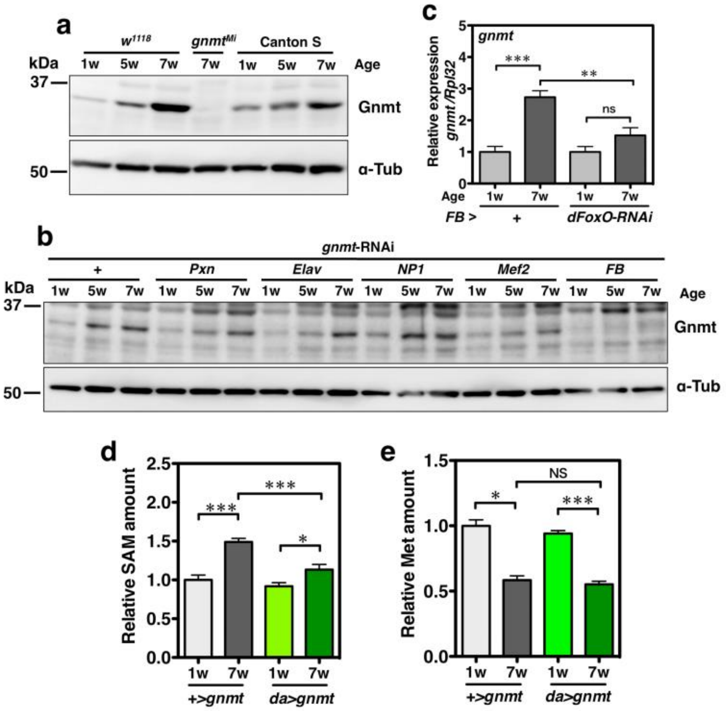

Supplementary Figure 4. Transcriptional upregulation of Gnmt by dFoxO in fat body in aged flies, and overexpression of *gnmt* rescued age-dependent SAM increase.

(a) Western blot analysis of Gnmt in wild type male flies during aging. $gnmt^{Mi}$ is used as a negative control. **(b)** Western blot analysis of Gnmt in *gnmt* knockdown male flies in various tissues. *UAS-gnmt*-RNAi flies were crossed to w^{1118} (negative control), or different Gal4 drivers, *Pxn* (hemocyte), *Elav* (neuron), *NP1* (enterocyte), *Mef2* (muscle), and *FB* (fat body). **(c)** qRT-PCR analysis of *gnmt* in *dFoxO* knockdown male flies. Error bars represent mean ± SEM (N=4). Statistics, two-tailed Unpaired *t*-test. (**d,e**) SAM and Met levels in young (1 week old) or old (7 week old) male flies. Metabolite levels were analyzed in *gnmt* overexpressing flies with (*da>gnmt*) or without ubiquitous driver, *da-Gal4* (Control).

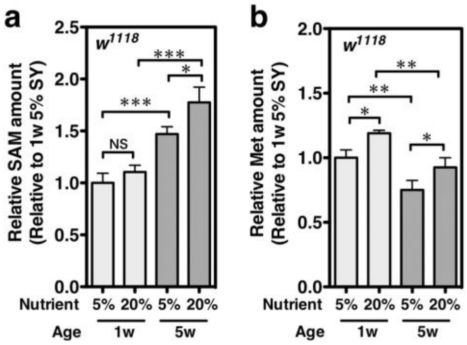

Supplementary Figure 5. Dietary restriction suppressed age-dependent SAM increases in *w*^{1118}

(a,b) SAM and Met levels in *w*^{1118} young (1-week-old) or old (5–week-old) male flies maintained under dietary restriction or nutrient rich condition. Error bars represent mean ± SD (N = 3–4). Statistics: One way ANOVA with Bonferroni's multiple comparison test.

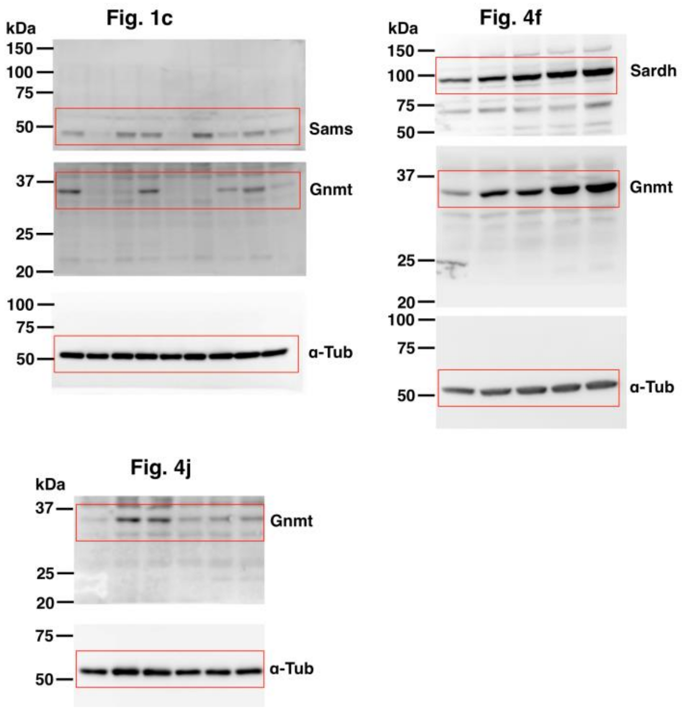

Supplementary Figure 6. Full blot images for western blotting in main figures

Supplementary Table 1. Primer sequences for qRT-PCR analysis

Target gene	Forward primer	Reverse primer
gnmt	5’- ctagacgtggcctgtggaac	5’- gcatctacggacacaacttcg
inr	5’- gcaaactctgccagacgaa	5’- cgcatccacccaaacaat
sardh	5’- acagggatcccagcaaaac	5’- gtcacatccgcctccact
sams	5’- gccaacggcgttcatatc	5’- ggcatatccaaacatgataccc
thor	5’- ccagatgcccgaggtgta	5’- agcccgctcgtagataagttt
gstD2	5’- ctccaatgtctccaggtggt	5’- cccagttctcatcccatcc
Rpl32	5’- cggatcgatatgctaagctgt	5’- cgacgcactctgttgtcg
RNApol2	5’- ccttcaggagtacggctatcatct	5’- ccaggaagacctgagcattaatct
